# The Well-Woman Project: Listening to Women's Voices

**DOI:** 10.1089/heq.2018.0031

**Published:** 2018-12-26

**Authors:** Arden Handler, Vida Henderson, Regan Johnson, Cristina Turino, Megan Gordon, Megan Franck, Nadine Peacock, Denise Pecha

**Affiliations:** ^1^ Community Health Sciences, University of Illinois School of Public Health, Chicago, Illinois.; ^2^ Cancer Center, University of Illinois, Chicago, Illinois.; ^3^ CityMatCH, University of Nebraska Medical Center, Omaha, Nebraska.; ^4^ CountyCare, Cook County Health and Hospitals System, Chicago, Illinois.; ^5^ Department of Epidemiology, University of Illinois School of Public Health, Chicago, Illinois.

**Keywords:** preventive care, well women, well-woman visit, women's voices

## Abstract

**Purpose:** The U.S. Affordable Care Act (ACA) of 2010 included the Well-Woman Visit (WWV) as one of the preventive services, which must be covered without cost sharing. Despite concerted efforts to increase access to the WWV, data from the early years of the ACA demonstrated ongoing barriers, including insufficient consumer and provider awareness of the ACA's no cost-sharing provision for preventive services. As such, 2 years after full implementation of the ACA, the Well-Woman Project (WWP) used qualitative methods to learn about women's perceptions of the WWV and barriers that affect their ability to be healthy and seek well-woman care.

**Methods:** Women's voices were captured by Listening Sessions in eight cities and through stories from women across the United States posted to a WWP Website, or reported over a WWP toll-free phone line. Thematic analysis of Listening Sessions and stories was conducted using Dedoose software.

**Results:** In 2016, Listening Sessions (17) were held with 156 women; in addition, stories were collected from 102 women across the United States. Women are aware of the importance of preventive care, but report multiple barriers to seeking such care. However, they are able to articulate a variety of system and policy strategies that mitigate the complexity of navigating the health care system; help women prioritize their health and accessing health care; promote positive relationships with providers; empower women to advocate for themselves and others; promote positive mental health as well as access to safe environments, healthy food, and social support systems; decrease barriers related to lack of transportation and childcare; and support the provision of trauma informed care in the health care delivery system.

**Conclusion:** To improve women's health status and reduce inequities, making the preventive well-care visit available without cost-sharing is necessary, but not a sufficient strategy.

## Introduction

The Affordable Care Act (ACA), passed in the United States in 2010 to increase access to health care for millions of uninsured Americans, included the Well-Woman Visit (WWV) as one of the preventive services which private health plans must cover without cost-sharing and which also must be covered for individuals accessing Medicaid through the ACA's Medicaid expansion.^1^ Expanded access to and use of the WWV have increasingly been seen as key strategies for promoting women's overall health and wellness throughout the life course, resulting in healthier women before, between, and beyond pregnancy.^2,3^

Despite concerted efforts over the last several years to increase access to the WWV, data from the early years of ACA implementation demonstrated ongoing barriers, including insufficient consumer and provider awareness of the ACA's no cost-sharing provision for preventive services.^4,5^Although 82% of women reported receiving a recent WWV in the 2013 Kaiser Women's Health Survey of a nationally representative sample of U.S. women 15–64 years of age, only 60% of women knew that insurance plans were required to cover WWVs.^4^ While there were no differences in WWV access in 2013 by race/ethnicity, uninsured women, low-income women, and those in poorer health were less likely to report having had a recent checkup with a provider.^4^ Although increasing utilization of the WWV is a key strategy for improving the health of women of reproductive age, it is also recognized that upstream drivers are main contributors to health status and health inequities.^6,7^

In 2016, 2 years into the full implementation of the ACA, the University of Illinois at Chicago School of Public Health (UIC-SPH) and CityMatCH, with funding from the W.K. Kellogg Foundation, launched the Well-Woman Project (WWP). Although multiple prior studies addressed women's barriers to health care, including barriers to primary and preventive care,^8–17^ the WWP sought not only to gather women's views about barriers to well-woman care access and the conditions of women's lives that affect their ability to be well-women and seek well-woman care, but also to gather their insights and explore the system and policy changes that are necessary to reduce inequities in women's health and health care.

## Methods

The stories of women of reproductive age were gathered through: (1) Listening Sessions (LS) held in conjunction with Maternal and Child Health programs (MCH) in health departments in eight cities (Boston, Chicago, Detroit, Jackson, Nashville, New Orleans, Oakland, and Omaha); (2) a toll-free 24/7 VOIP Story Phone line (in Spanish and English); and (3) a confidential WWP Story Blog/Website. The Story Phone line and Blog/Website were available to women across the eight cities as well as nationally.

The eight cities were selected in conjunction with the W. K.Kellogg Foundation and CityMatCH based on whether a city and its MCH leaders were active CityMatCH participants and/or whether the city hosted other Kellogg projects. The LS and Story/Blog websites/phone line were advertised through the health departments' and/or the authors' multiple networks and partners using a Twitter account, magnets, memes, and flyers. Flyers and memes were available in English and Spanish (e.g., Tell us Your Story: What does being Healthy Mean to you?) and incorporated multicultural tailored images.

The LS were guided conversations in English or Spanish (if the majority of women in the group were not native English speakers as Spanish was the other major language of participants) facilitated by research team members; at least two were held in each city. The LS guide sought to gather women's thoughts, beliefs, and experiences with respect to use of preventive care, WWV utilization and access, and conditions of their lives that affect their ability to be well-women. In exchange for their participation, women received a stipend. All conversations were recorded, transcribed, and analyzed using Dedoose qualitative analysis software.

There were two WWP secure and confidential Story Blog/websites, one in English and one in Spanish, where women could post their Stories related to their health and health care. The Story Phone Line was a secure and confidential toll-free phone line; women could share their Stories in either English or Spanish. Audio files of Stories were automatically sent to a private e-mail address, and all data from both the Blogs and Phone Line were downloaded, transcribed, and analyzed by UIC researchers using Dedoose software.

Analysis of qualitative data from transcripts of the Listening Sessions and Stories was conducted through a process of thematic analysis. The approach to analyzing the LS and the Stories was the same. First, all team members participated in review and annotation of the transcripts. The annotations were used to develop a set of codes that identified key concepts in the data; related codes were grouped into code families. Two team members (V.H., M.G.) independently coded a subset of transcripts, and percent agreement was assessed as a measure of intercoder reliability. Discrepancies were discussed and resolved, followed by another round of independent coding. Once the two coders reached a percent agreement of at least 90% (this occurred after three rounds of individual coding), they divided the remaining transcripts and each coded a set. Codes and code families were used to extract related passages of data, which were then examined to identify common themes.

The themes generated were based on commonality of ideas and opinions expressed by women in the Listening Sessions in each respective city, across all cities, and across the shared Stories; no theme was based on comments from just one or two women. While initially, the Stories and Listening Sessions were analyzed separately, the commonality of overarching concepts that emerged led the researchers and project personnel to combine the data from both sources. Recommendations were then generated through an iterative process and comprised ideas that came from the following: (1) the women who shared their experiences; (2) the research team's partners/stakeholders; and (3) the research team itself.

In Winter/Spring 2017, overall and city-specific recommendations were shared with each health department and were incorporated into city-specific profiles on the status of women's health. (Note: Profiles are not shared here). This project and all of the various materials were approved by the University of Illinois at Chicago IRB.

## Results

During the Spring of 2016, 156 women participated in 17 LS in the 8 participating cities, with an additional 102 Stories collected through the Phone Line (*n*=4) and Blogs (*n*=98). Women participating in the LS were mostly African American (AA) and Latina and had Medicaid coverage for insurance; their mean age was 28.8. Most were not working, although over 45% were college educated (Table 1). With respect to the Blog and Phone Line Stories, we were only able to gather information on the ages and cities of the participating women; most women who contributed Stories were not from the eight LS cities and most were 18–35 years of age (Table 2).

**Table 1.** Demographic Characteristics of Well-Woman Project Listening Session Participants (*n*=155)

Demographic characteristics	*N*	Mean or percent
Age	152	28.8
Missing	3	
Race/Ethnicity		
Black non-Hispanic	95	61.3%
White non-Hispanic	8	5.2%
Hispanic	39	25.2%
American Indian/Alaska native	1	0.6%
Native Asian, native Hawaiian, or Other Pacific Islander	3	1.9%
Multiracial	8	5.2%
Missing	1	0.6%
Education		
Less than high school	33	21.2%
High school graduate	30	19.4%
Some college	30	19.4%
College graduate	21	13.5%
Graduate school	19	12.3%
Currently in school^a^	17	11.0%
Missing	5	3.2%
Number of living children	150	1.65
Employment status		
Full time	41	26.5%
Part time	26	16.8%
Not currently employed	82	52.9%
Missing	6	3.9%
Health insurance coverage		
Medicaid	91	58.7%
Private insurance	37	23.9%
Multiple insurance	6	3.9%
Other	4	2.6%
No insurance coverage	14	9.0%
Missing	3	1.9%
Obtained insurance through ACA?		
Yes	23	14.9%
No	95	61.7%
Don't know/Not sure	32	20.8%
Missing	4	2.6%

One participant in the Listening Sessions did not complete a demographic survey.

^a^ 15/17 in certificate program, trade school, college, and graduate school.

**Table 2.** Characteristics of Stories Collected through the Well-Woman Project (*n*=102)

Source of stories	
Website/blog	98
Phone	4
Language of stories	
English	101
Spanish	1
Location of stories (website and phone combined)	
Chicago	15
Omaha	6
Detroit	6
Oakland, CA	2
Boston	8
Nashville	3
New Orleans	1
Jackson, MS	2
Other	59
Age of story participants	
18–35	73
36–45	19
46–64	9
65+	0
Missing	1

Themes (discussed below Theme #1: the health care system is not women-friendly section) and relevant quotes are provided in Table 3. Themes are not presented by level of importance. Each theme illustrates factors that impact women's holistic experiences of health, health care, and well-being.

**Table 3.** Themes, Quotes, and Sample Recommendations Associated with Each Well-Woman Project Theme

Well-woman project theme	Representative quotes	Aim of recommendations	Sample recommendations- city MCH leaders to
Theme #1: The health care delivery system is not woman friendly.	*I have to go through this and that and the third or go to where they want me to go. So it becomes a very difficult process when you actually really do have a medical problem, you know? It makes it very difficult for you to get. And I think it's great that any person and any adult who does not have children can get health care. That's wonderful. But it's just a very nerve-wrecking process.* (Chicago, IL)	Strategies to make navigating the complex health care delivery system easier.	Engage in dialogue with both large health systems and FQHCs to encourage increased availability of appointments outside of traditional hours, drop-in/walk-in appointments, and online and/or phone consultation.^a^
Theme #2: Women's competing demands and priorities make accessing health care difficult.	*Competing priorities get in the way of my ability to be healthy. Between work, childcare, and home duties—there is little time left over for me to take care of myself. I worry about making sure that everyone else is well taken care of that I often leave myself out.* (Decatur, GA)	Strategies that allow women the appropriate time to take care of their needs.	Work with partners to create a city-wide task force to consider adoption of paid sick leave for public and private employees (if not available).^a^ For cities with sick leave policies, develop policy and educational materials for consumers focused on city sick/personal leave policies.
Theme #3: Women weigh cost vs. benefits when deciding to access care.	*I guess it's not necessarily that women don't think it's important. It's just it's too much. It's a hassle to figure out. You have to put too many things in place, and then, it's kind of like you're weighing the worth of it versus the cost of it. Is it worth it? Am I going to gain anything from putting everything else I have to do on hold to do it?* (New Orleans, LA)	Strategies to increase the transparency of the health care system and lower health care costs.	(1) Partner with major health systems, FQHCs, and other key stakeholders to provide women and families with access to insurance navigators on a year-round basis;^a^ (2) develop a city fund to cover uninsured women and families and/or help women and families struggling with high deductibles; (3) partner with major health systems/FQHCs to sponsor “One Day” Medicaid/free care for all events.^a^
Theme #4: Relationships with providers are key to women's decisions to access care.	*Building a relationship with your doctor - when you have that type of relationship, you don't have to run into things where they're just trying to get you out of the office because they have ten people sitting in the waiting room. You build that relationship to where they are calling you and asking you how you are. “Is it feeling better? Is it worse? Do you need to come in? Do you need to see a specialist?” When you have that relationship, that makes you feel a lot better.* (Detroit, MI)	Strategies to increase trust, comfort, and rapport between women and providers, including providers' staff.	(1) Explore approaches to the development of a women-centered*,* consumer-driven mechanism to enable women to review providers and recommend women-friendly provider sites;^a^ (2) partner with major health systems and FQHCs to develop and offer training to increase the cultural humility of the clinical workforce;^a^ and (3) explore approaches that enable women to have their health *histories* available on personal “apps” so that providers can readily access this information.
Theme #5: Health & insurance literacy empower women to advocate for themselves and others.	*I guess it's all about the information…And so asking and calling to make sure you get information and knowing about the different services and what you want because it's all about questions. If you call and ask, you're going to get your services how you want them*. (Boston, MA)	Strategies to increase access to health education; improving health literacy to empower women to advocate for themselves and others.	(1) Ensure the availability of a city-wide Women's Health Hotline as a go-to resource for up-to-date information on changing health and health care recommendations and guidelines;^a^ (2) provide trainings focused on how to advocate for one's self/family with both providers and insurance companies; and (3) provide updated lists of available providers, including the types of insurance policies they accept, as well as providers who offer free or sliding scale services.^a^
Theme #6: Positive mental health is integral to being a “healthy” woman.	*As an African-American woman I have encountered many traumatic experiences such as rape, physical abuse, and verbal abuse. All of which have taken a toll on my mental health. I learned early in life that seeking psychological counseling is not acceptable in the black community. But as I got older, I realized how important it is to make sure my entire body is healthy, including my mind and emotional state. So I sought out counseling over twenty years ago.* (Oakland, CA)	Strategies focused on improving access, affordability, and social acceptability of mental health care.	(1) Work with community partners to ensure the availability of community-based resources for self-care and respite; (2) partner with major health systems and FQHCs to increase care coordination between mental health and primary health care providers; and (3) educate communities about the importance of mental health care to reduce stigma.
Theme #7: Healthy food, safe environments, and opportunities for physical activity are vital for women.	*Let's not forget the crazy costs of healthy foods, either. Our frugal family manages to get most of the health foods we want/need, but we do so by shopping sales mainly. That's a tough one when Walmart is the only option for 10–15 miles. Otherwise, we're talking extra money for gas to grocery shop too*. (Hiawatha, KS)	Strategies to improve access to affordable quality food and safe and affordable environments for daily living and routine physical activity.	(1) Explore “food prescription” approaches and/or community-supported agriculture (CSA) programs; (2) explore ways to improve women's and families' abilities to apply for SNAP at their health care providers' offices; and (3) partner with groups such as the YMCA/YWCA to provide low-cost gym memberships;^a^ (4) create “safe” walking and jogging paths; and (5) partner with mental health providers to offer trauma-informed care for women who have experienced violence in their homes and/or neighborhoods.
	*In my household of violence, surviving was a more important priority than staying healthy. Rarely did my mom and sister and I get to discuss health challenges, especially mental health since we were trying to stay alive most of the time. Years, later, even after my abuser has died, I am trying to piece together my health again. It is challenging. I wish I had a head start on all of these things instead of learning about them after the fact.* (Roxbury, MA)		
Theme #8: Social support systems facilitate women's willingness and ability to seek care.	*So going alone, you can have a fear or an intimidation factor as well, especially if you're being given a diagnosis, not just for general treatment that you don't have any problems, really. And so having someone with you that you trust, a relative, friend, child, or whatever, it can help you as well. And older people, and people that are very young, just starting out, many times, they don't have that security, they don't have the knowledge, they don't have that confidence.* (Jackson, MS)	Strategies to promote personal and system-facilitated social support networks to increase women's willingness and ability to seek care.	Explore the development of a cadre of women's health peer advocates (volunteer or paid) who can be present at women's health appointments and work with health systems, FQHCs, and other stakeholders to increase “group” approaches to care.
Theme # 9: Fear is a pervasive component of many women's health care experiences.	*That's probably why a lot of people don't wanna go because if you get your results, you're scared and you're panicking, so that's probably why a lot of people don't wanna go to the doctor. They're scared of the results.* (Jackson, MS)	Strategies to reduce women's sense of fear and trauma in their health care experiences.	Work with partners and health systems to encourage and support the provision of training in trauma-informed care for health care providers.
	*I have a history of sexual trauma, and going to my GYN for regular checkups is a nightmare for me. I can't handle it, so I avoid it altogether.* (Syracuse, NY)		
Theme #10: Lack of childcare and transportation are major impediments to accessing health care.	*As far as transportation, the vans that Medicaid pays for to take people, they should work more with the patient because now they don't even provide rides to the WIC office and they won't let your other kids ride with you unless they have an appointment.* (Omaha, NE)	Strategies to improve transportation provided through Medicaid/insurance and increase the accessibility of public transportation for women and children as well as easing the mental and logistical burden for women with children to enable them to attend visits and obtain well-woman care.	(1) Partner with ride-sharing organizations to transport patients and their families to and from their medical appointments;^a^ (2) provide free parking vouchers or free or discounted bus/train cards to attend appointments;^a^ (3) provide play areas or supervised childcare facilities in their clinics/offices;^a^ and (4) engage with Dept. of Transportation to explore plans to provide women and child-friendly public transportation.^a^
	*So I've skipped an appointment because I say how are they going to be examining me with two children there? I don't have any place to leave them.* (Oakland, CA)		

^a^ Indicates recommendations that women directly provided; however, other recommendations are also informed by the experiences and opinions shared by women.

Because the project was conducted in conjunction with eight city health departments, city-level recommendations were developed. These were framed as policy or system-level recommendations that might be addressed by MCH leaders in cities, but most have relevance beyond the cities included in this project. Examples of these recommendations are also presented in Table 3; column 3 of this table presents the focus of the recommendations for each theme,while column 4 presents sample recommendations. Recommendations directly provided by women through the LS or Story Blogs/phone line are noted with an asterisk.

### Theme #1: the health care system is not women-friendly

Women reported that health care costs and system and insurance barriers often prevented them from obtaining health care. Even with the insurance and Medicaid expansions of the ACA, women faced barriers in obtaining any or low-cost insurance and reported issues with copayments, deductibles, and premiums. Many avoided seeking health care because they were afraid they could not afford the associated costs. Women also reported difficulties related to making appointments, the office wait times associated with appointments, and adhering to the referral requirements of their insurance policies.

### Theme #2: women's competing demands and priorities make accessing health care difficult

Women expressed that preventive care is very important to maintaining their health; however, they also expressed difficulties in prioritizing care due to a lack of resources and information. Many stated that the competing demands of their work, family, and home duties often prevented them from engaging in healthy behaviors as well as from seeking care. Women also discussed having jobs that do not offer paid sick time, personal days, or vacation time to see a health care provider, leaving them unable to make appointments during traditional office hours.

### Theme #3: women weigh costs versus benefits when deciding to access care

Because of the complexity of the health care system, the potential for hidden and unknown costs, difficulty in accessing care, and competing demands and priorities, women expressed the need to weigh the worth of rearranging their schedules, finding child care (if needed), paying for transportation, leaving work early, and facing cost uncertainty against the benefits of seeking care, particularly when not sick.

### Theme #4: relationships with providers are key to women's decisions to access care

An important facilitator of women seeking preventive care is having relationships with providers whom they trust. However, women made it clear that they do not always feel heard, and that many providers do not address their concerns. Many also expressed being discriminated against due to their race or ethnicity, socioeconomic status, type of insurance, disability, and sexual orientation or gender. Spanish-speaking women often noted a lack of translational services and materials available in Spanish, which made communication with providers difficult.

One factor that promotes trust is having the same provider over time who is familiar with the woman's medical history. However, this has become more of a challenge as women change insurance networks when they move, transition between insurance plans, or change employment.

### Theme #5: health & insurance literacy empower women to advocate for themselves and others

A lack of health and/or insurance knowledge or literacy also serve as barriers for many women. Women who were knowledgeable about preventive care services were aware of specific screening recommendations, but were often confused about timing. Many women, particularly younger women, expressed not being educated about their health or their bodies, so preventive care was an unfamiliar concept.

### Theme #6: positive mental health is integral to being a “healthy” woman

Women identified that stress and trauma cause ill health and stated that women with positive mental health are more likely to be well-women. While women valued the importance of mental health, many described barriers to accessing mental health services. Women also noted that the perceptions and beliefs held by family and friends affect how frequently they sought care for psychological concerns and that using mental health services is often stigmatized.

### Theme #7: healthy food, safe environments, and opportunities for physical activity are vital

Women recognized that factors external to the use of health care affected their health and well-being. Although many expressed a desire to eat and cook healthy meals, they identified multiple barriers preventing them from doing so, including costs and time. Likewise, some women identified that the nonwalkability of their neighborhoods prevents them from engaging in exercise or spending time outdoors. In addition, living and/or working in high crime neighborhoods and/or unsafe home environments often cause women to consider how to *survive* in their current living situations, rather than how to *thrive* and improve their health and well-being.

### Theme #8: social support systems facilitate women's willingness and ability to seek care

Women expressed the desire for social support when visiting health care providers as well as the need for assistance with everyday tasks and responsibilities to ease pressure. Many recognized that the lack of emotional and physical support affected their ability to be healthy.

### Theme # 9: fear is a pervasive component of many women's health care experiences

Given an expressed lack of health knowledge or literacy, women expressed fear about multiple issues. In addition to fear related to finances, several expressed fear about being judged or stigmatized for utilizing care and/or fear about the content or results of the visit. Many reported fear related to lack of citizenship or immigration status, while some feared the loss of confidentiality.

### Theme #10: lack of childcare and transportation are major impediments to accessing health care

Multiple women reported that barriers related to transportation and childcare affected their ability to obtain care. Women in many cities reported long distances to providers, no available parking, and unreliable and unsafe public transportation when traveling with small children, including no room for car seats or strollers on buses, in particular. Women also discussed unreliable transportation services that are not family friendly. Several reported being unable to take their children to their appointments due to a lack of child-friendly clinics and/or being unable to obtain child care to attend their health care appointments.

In addition to the across-study themes discussed above, city-specific themes also emerged. Women reported differential treatment based on their race or ethnicity in four cities, differences in access to and quality of care based on insurance status in five cities, and difficulty or fear in accessing care if they did not speak English or were not citizens in six cities. In six cities, women reported that pregnancy was their introduction to the health care system as Medicaid coverage in many states is still focused on categorical funding, which prefers payment for pregnancy care over nonpregnancy care.^18^

## Discussion

The WWP aimed to gather women's views and stories with respect to the WWV and the conditions of their lives that affect their ability to be well-women and seek well-woman care. This is important as a recent study found that despite the ACA's emphasis on preventive care, “forty percent of women are unaware that they are entitled to WWVs under the ACA and one in five women postpone preventive care owing to cost.”^5^ Through the elevation of their voices, women in the WWP not only acknowledged the importance of preventive care but also elucidated the multiple barriers to seeking and obtaining such care, including personal, provider, and system factors. Based on the results reported in this study, it appears that to improve women's health status and reduce inequities, making the preventive WWV available without cost-sharing is a necessary, but not sufficient strategy.

With respect to personal factors, prior research has shown that lower socioeconomic status acts as a substantial barrier to women utilizing preventive services.^8–10^ In addition, women with higher education, high income, and who are employed are more likely to use preventive services.^10–14^ In this study, the majority of women were on Medicaid and not employed; although their direct utilization of preventive care was not measured, they faced multiple barriers to care related to the competing demands of their lives.

Interestingly, one prior study found women with high levels of familial social support less likely to pursue preventive care. However, in that study, as social support from friends grew, so did the likelihood they would utilize preventive care, seemingly because the indirect experiences women have with preventive care through their peers increase their probability of use.^15^ Women in this study indicated that social support was an important factor in their willingness to use preventive care.

Most of the provider and system factors identified in this study have been noted previously, including not having a usual source of care and/or lacking health insurance.^9,10,12,13^ Independent of insurance, women in prior studies have reported other barriers to care such as inconvenient office hours, competing demands, and lack of childcare and transportation,^16,17^ all of which were elucidated in this study. In addition, in a study on prenatal care, women described differential treatment based on type of health insurance and on race/ethnicity,^14^ phenomena also reported in this study. A recent post-ACA implementation study, found that primary care providers are ordering more preventive services for women with private insurance than for women with Medicaid, thus further perpetuating disparities that already exist based on socioeconomic factors.^19^ As also noted in this study, prior literature has shown that women prefer providers who are nonjudgmental, trusting, and good listeners as well as those who display empathy and encourage patient engagement in their care and use of services.^16^

The LS and Stories revealed that even though women may understand the importance of prevention, they were often confused about recommendations related to seeking preventive health care (Theme #5). According to Pascale et al.^20^ in their review of the relevant content for the WWV: “At the same time that health plans are implementing the ACA-mandated coverage for annual WWVs without cost sharing, certain key components of the traditional WWV, such as the annual Pap smear for cervical cancer screening and the bimanual pelvic examination, are being challenged.” Pascale et al. suggest that new recommendations have led to “headlines in the popular press and professional editorials, which question the need for any kind of preventive care at all” (p.136). However, they conclude that the “WWV remains a very important opportunity for prevention, health education, screening, and early detection” (p.135).

A unique contribution of this study is that it goes beyond previous studies that typically document factors associated with and barriers to preventive and well-woman care, and proposes a series of system and policy recommendations that city MCH leaders in particular can undertake to reduce inequities in women's use of health care and health status outcomes. That many were generated by the women themselves speaks to the value of attending to consumer voices in the development of patient-centered and woman-friendly programs and policies.

Since increasing access to the WWV is a priority for the 50 state Title V/MCH programs across the nation,^21^ a deeper exploration of these recommendations is needed. Many have been implemented in one form or another in various locales or within individual health systems, clinics, or organizations; however, an evidence review for those that are of interest to individual locales should be undertaken. Given the volume of recommendations, such a review was not undertaken in this study. In addition, attempts by individual cities and other locales to implement any of these recommendations might benefit from an implementation science approach.^22^

Despite the richness of the information gathered, there are several limitations. This is a qualitative study aimed at garnering a deeper understanding of women's lived experiences in relationship to preventive care utilization and access; as such, the participants are not a representative sample of women in the eight cities or the nation. In each city, while recruitment materials were the same, strategies for recruiting women to the LS varied slightly and although the focus of the LS was on women of reproductive age, there were no other criteria. Because MCH programs in city health departments recruited the women, the majority were low-income and mainly Medicaid recipients. In addition, although not by design, the health departments were predominantly in communities with high concentrations of AA; therefore, the majority of LS participants were AA. With respect to the Stories, women came from all eight cities as well as from 58 additional locales. Unfortunately, the only demographic information available about these women is their age, with the majority reporting themselves to be between 18 and 35 years of age (similar to the mean age of the women in the LS), in line with the focus on reproductive aged women. In addition, because the themes were integrated across the LS and Stories, any unique themes that might have emerged from one or the other source are not explicated in this study. Despite these limitations, we believe this study has generated important information for efforts to improve women's health and to increase access to well-woman care.

## Conclusion

The results of the WWP suggest that efforts to increase the use of well-woman care and reduce inequities across class and race/ethnicity in women's health status outcomes will not be successful if the focus is only on the use of care without considering the context of women's lives, and if only traditional approaches to increasing access such as education and media campaigns are utilized. Rather, attention to a host of other factors addressing issues as varied as the structure of the health system, women's fears about using care, and transportation and childcare will be necessary. As multiple strategies to address all of these issues are available, moving forward, policy-makers and health care professionals will need to select, modify, implement, and test these strategies to increase women's access to preventive care and to increase their opportunity to be well-women.

## Abbreviations Used

ACA: affordable care act
MCH: maternal and child health programs
WWP: well-woman project
WWV: well-woman visit
